# BRAF and MEK inhibition in melanoma patients enables reprogramming of tumor infiltrating lymphocytes

**DOI:** 10.1007/s00262-020-02804-4

**Published:** 2020-12-04

**Authors:** Lukas Peiffer, Farnoush Farahpour, Ashwin Sriram, Ivelina Spassova, Daniel Hoffmann, Linda Kubat, Patrizia Stoitzner, Thilo Gambichler, Antje Sucker, Selma Ugurel, Dirk Schadendorf, Jürgen C. Becker

**Affiliations:** 1 Deutsches Konsortium Für Translationale Krebsforschung (DKTK), Partner Site Essen, Translational Skin Cancer Research, University of Duisburg-Essen, Universitätsstr. 1, 45141 Essen, Germany; 2 Deutsches Krebsforschungszentrum (DKFZ), Heidelberg, Germany; 3 Bioinformatics and Computational Biophysics, University Duisburg-Essen, Essen, Germany; 4 Department of Dermatology, Venereology and Allergology, Medical University of Innsbruck, Innsbruck, Austria; 5 Department of Dermatology, Skin Cancer Center, Ruhr-University Bochum, Bochum, Germany; 6 Department of Dermatology, University Hospital of Essen, Essen, Germany

**Keywords:** BRAF/MEK inhibition, Tumor microenvironment, T-cell receptor repertoire, TCF7, MDA, CTA

## Abstract

**Background:**

Combined inhibition of BRAF/MEK is an established therapy for melanoma. In addition to its canonical mode of action, effects of BRAF/MEK inhibitors on antitumor immune responses are emerging. Thus, we investigated the effect of these on adaptive immune responses.

**Patients, methods and results:**

Sequential tumor biopsies obtained before and during BRAF/MEK inhibitor treatment of four (*n* = 4) melanoma patients were analyzed. Multiplexed immunofluorescence staining of tumor tissue revealed an increased infiltration of CD4^+^ and CD8^+^ T cells upon therapy. Determination of the T-cell receptor repertoire usage demonstrated a therapy induced increase in T-cell clonotype richness and diversity. Application of the Grouping of Lymphocyte Interactions by Paratope Hotspots algorithm revealed a pre-existing immune response against melanoma differentiation and cancer testis antigens that expanded preferentially upon therapy. Indeed, most of the T-cell clonotypes found under BRAF/MEK inhibition were already present in lower numbers before therapy. This expansion appears to be facilitated by induction of T-bet and TCF7 in T cells, two transcription factors required for self-renewal and persistence of CD8^+^ memory T cells.

**Conclusions:**

Our results suggest that BRAF/MEK inhibition in melanoma patients allows an increased expansion of pre-existing melanoma-specific T cells by induction of T-bet and TCF7 in these.

**Electronic supplementary material:**

The online version of this article (10.1007/s00262-020-02804-4) contains supplementary material, which is available to authorized users.

## Introduction

Combined BRAF/MEK small molecule inhibition is an established therapy for BRAFV600E-mutant melanoma [1]. However, the effects of these small-molecule inhibitors on the immune system, particularly on T-cell responses, are not fully understood. Since T-cell activation via the T-cell receptor (TCR) and its costimulatory molecules depends on the mitogen-activated protein kinase (MAPK) and PI3K-AKT signaling cascades [2], MEK inhibitors may impair T-cell activation. Indeed, several reports demonstrated that the pharmacologic inhibition of MEK in vitro has detrimental effects on T cells [3–5]. In contrast, in vivo analyses showed both an improved activity of adoptive T-cell transfer with no adverse effects on the T-cell effector functions, but also favorable results in combination with immune checkpoint inhibition [6–8]. MEK inhibition was associated with an increase in CD8^+^ T-cell infiltration of tumors, elevated interferon-gamma (IFN-γ) gene expression signatures, as well as a decreased presence of tumor-associated macrophages and regulatory T cells [6–8]. In addition, effector T cells are protected by MEK inhibition from activation-induced cell death caused by chronic TCR stimulation [7]. Indeed, comparison of antitumor effects of BRAF/MEK inhibition in immunocompetent and immunocompromised mice revealed the importance of immunoregulatory effects, as immunocompetent mice showed a significantly longer duration of response [9]. Thus, the combination of BRAF/MEK and immune checkpoint inhibition is currently tested in various clinical trials. First results of these triple combination trials are promising. Larger clinical trials testing either the sequential application of targeted and immune therapy (NCT03149029, NCT02858921, NCT02625337) or their combination (NCT02902042) are ongoing. First positive results have been reported for combination of dabrafenib, trametinib, and pembrolizumab (NCT02130466) [10] and of atezolizumab, vemurafenib and cobimetinib (NCT02908672) [11], which was approved by the FDA in July 2020.^1^

Here, we scrutinized the impact of BRAF/MEK inhibition on adaptive immune responses in melanoma patients by performing a comprehensive immunological characterization in sequential tumor biopsies obtained before and during BRAF/MEK inhibition.

## Materials and methods

### Patients

**Fig. 1:**
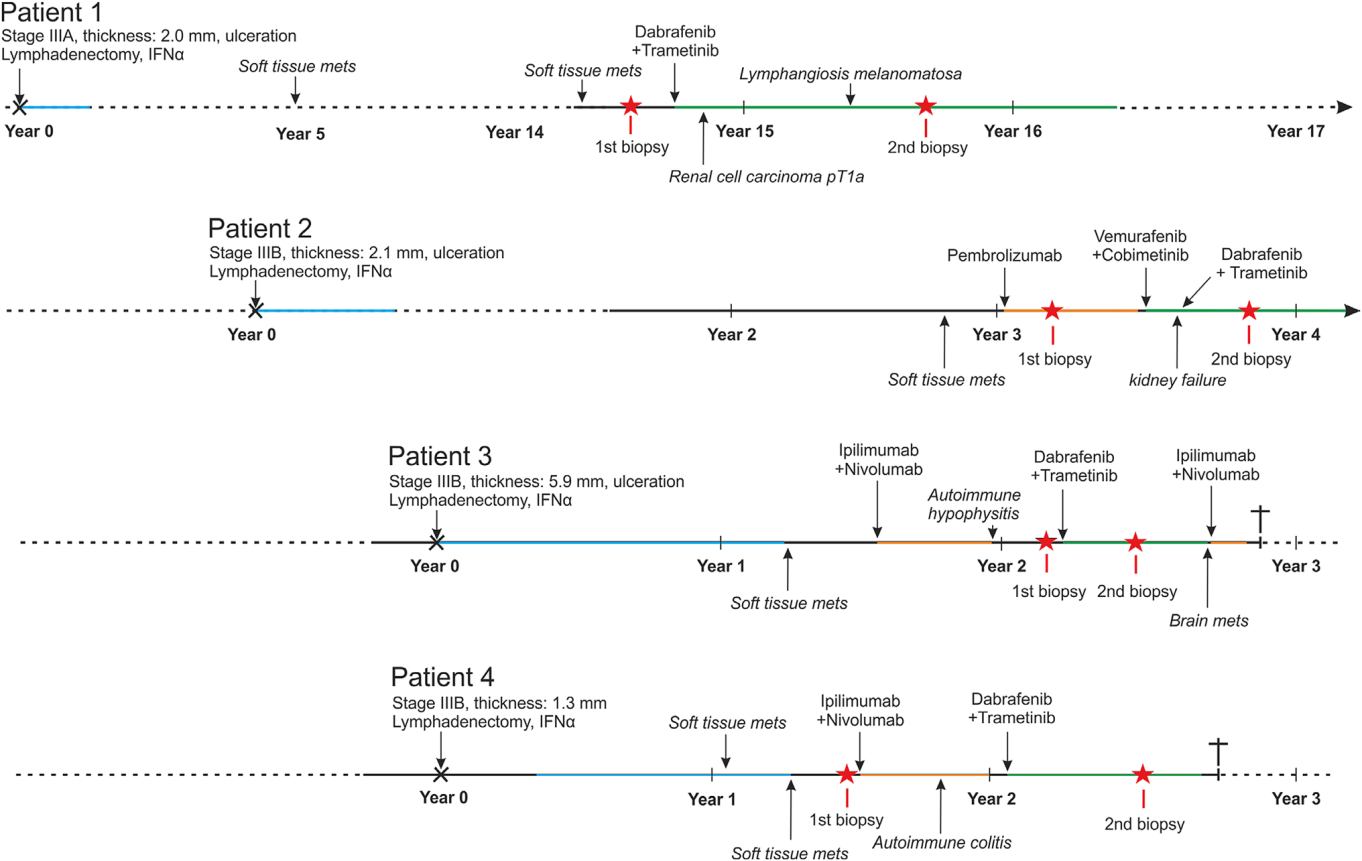
Patients’ history. The melanoma-specific history is provided for the four investigated patients. Disease stage according to AJCCv8 and therapeutic procedures at first diagnosis are given at year 0. Prior systemic therapies include adjuvant IFN-α (blue) and therapeutic immune checkpoint inhibition (orange). Duration of therapeutic BRAF/MEK inhibitor administration is marked in green. Red asterisks indicate the time points of tumor tissue biopsy. Although two patients responded to BRAF/MEK inhibition and are still alive, the other two showed no response and rapidly succumbed to the disease (indicated by a cross)

Biopsies of metastatic lesions were obtained from four patients with histologically confirmed nonresectable metastatic melanoma before and during BRAF/MEK inhibitor therapy (150 mg dabrafenib twice, 2 mg trametinib once per day orally) at the Department of Dermatology, University Hospital Essen, Essen, Germany. Tumor stage was classified according to AJCCv8 [12]. The presence of the BRAF V600E mutation was confirmed by targeted next-generation sequencing prior to treatment. The patients’ melanoma-specific history and clinical details are given in Fig. 1 and Table 1.

**Table 1:** Patient and tumor characteristics

Patient	Primary tumor site; invasion depth; stage at diagnosis	Prior therapies	BRAF/MEK inhibition therapy	1^st^ biopsy: stage at that time	Best response to BRAF/MEK inhibition	2^nd^ biopsy: stage at that time
1	Lower leg, 2.0 mm; IIIC pT2b pN1a cM0	IFNα	dabrafenib + trametinib	Skin metastasis (in transit); IIIC pT2b pN2c cM0	PR	Skin metastasis (in transit), regressing upon therapy; IIIC pT2b pN2c cM0
2	Lower leg, 2.1 mm; IIIC pT3b pN1a cM0	IFNα, pembrolizu-mab	vemurafenib + cobimetinib; dabrafenib + trametinib	Skin metastasis (in transit); IIIC pT3b pN2c cM0	CR	Skin metastasis (in transit), regressing upon therapy; IIIC pT3b pN2c cM0
3	Lower leg, 5.9 mm; IIIC pT4b pN1a cM0	IFNα, nivolumab + ipilimumab	dabrafenib + trametinib	Skin metastasis (in transit); IIID pT4b pN3c cM0	PD	Skin metastasis (in transit), progressing upon therapy; IIID pT4b pN3c cM0
4	Thorax, 1.3 mm; IIIB pT2b pN1a cM0	IFNα, nivolumab + ipilimumab	dabrafenib + trametinib	Skin metastasis (in transit); IIIC pT2b pN2c cM0	PD	Lymph node metastasis, progressing upon therapy; IIIC pT2b pN2c cM0

Tumor staging was classified according to AJCCv8

*PR* partial response, *CR*,complete response, *PD* progressive disease

### Multiplexed immunofluorescence

Formalin-fixed paraffin-embedded (FFPE) tissue sections were analyzed by multiplexed immunofluorescence staining for CD4, CD8, CD20, CD68 and FOXP3 expression using the Opal-7® Solid Tumor Immunology Kit (Akoya Biosciences, Marlborough, MA/Menlo Park, CA, USA) according to manufacturer’s instructions. An additional custom panel was established to stain for CD8 (SP16, Biocare Medical, Pacheco, CA, USA; 1:100, 30 min), TCF7 (C63D9, Cell Signaling, Danvers, MA, USA; 1:100, 30 min) and granzyme B (GrB) (ab4059, Abcam, Cambridge, UK; 1:100, 30 min).

After deparaffinization and fixation, 3 µm sections were processed with retrieval buffers for 15 min in an inverter microwave oven. Thereafter, sections were incubated with the antibody diluent for 10 min at room temperature, followed by incubation with the primary antibody for 30 min. After applying Opal Polymer horseradish peroxidase (HRP) secondary antibody solution for 10 min, antibodies were removed by microwave treatment before the next round of staining. Additionally, sections were stained with an antibody against MART-1 (MSK056, Zytomed, Berlin, Germany) at a concentration of 1:100 for 30 min at room temperature. At the end, sections were incubated with DAPI for 5 min. Visualization of the different fluorophores was achieved on the Mantra Quantitative Pathology Imaging System (Akoya Biosciences, Marlborough, MA/Menlo Park, CA, USA). Multispectral images were analyzed with the Quantitative Pathology Imaging System Software inForm (Akoya Biosciences, Marlborough, MA/Menlo Park, CA, USA). As a first step, autofluorescent background was removed. Subsequently, cell segmentation algorithms and marker positivity were established on a representative section of each patient to apply it to at least 4 different areas of the tumor lesion (20 × magnification), based on which the average infiltration was calculated.

### Quantitative real-time PCR (qRT-PCR)

RNA was extracted using the AllPrep DNA/RNA FFPE Kit (Qiagen, Hilden, Germany) and transcribed into cDNA with SuperScript IV reverse transcriptase according to the manufacturer's instructions. qRT-PCR was performed on the CFX Real-Time PCR system (Bio-Rad Laboratories, Hercules, CA, USA). For the detection of T-bet and TCF7 expression using SYBR green assays, RPLP0 was used as endogenous control. The following relative quantification was done by the 2− ΔΔCq method. Primer sequences are given in Suppl. Table S4.

### TCRβ complementarity determining region 3 (CDR3) analysis by high-throughput sequencing

Genomic DNA was extracted from FFPE tissue with the AllPrep DNA/RNA FFPE kit (Qiagen, Hilden, Germany). Amplification and sequencing of the CDR3 of the different TCRβ families was performed using the ImmunoSeq™ (Adaptive Biotechnologies, Seattle, USA) protocol. In brief, highly optimized multiplexed PCR primers were used to amplify the respective CDR3s. Universal adaptor sequences and DNA barcodes were added by a second PCR run before high-throughput sequencing using the MiSeq ReagentKit v3 150-cycle in a MiSeq system (Illumina, San Diego, CA, USA).

### Statistical and bioinformatics analyses

Several statistical measures were used to describe dynamics of the TCR repertoire: (1) Observed richness is the number of unique nucleotide rearrangements in the sample; (2) estimated richness as calculated by iChao1 is an estimator for the lower bound of clonotype richness [13]; (3) Simpson’s diversity (Simpson’s D), the probability that two T cells taken at random from a specimen represent the same clone, is calculated as the sum over all observed rearrangements of the square fractional abundances of each rearrangement [14]. GraphPad Prism 5 (GraphPad Software, San Diego, CA, USA) was used to perform the statistical tests. Two-tailed Student’s *t* test was used to compare before and under therapy with *P* values < 0.05 considered as statistically significant.

The Grouping of Lymphocyte Interactions by Paratope Hotspots (GLIPH) algorithm was applied to reveal TCR CDR3s with similar antigen specificities. The algorithm clusters CDR3 amino acid sequences according to their local and global similarity [15]. A local similarity exists if two sequences contain the same specific motif of 3 or 4 amino acids, which is overrepresented in the respective data set compared to a reference database. A global similarity is assumed if two sequences have a Hamming mutation distance of one. The algorithm was run with default parameters. To estimate the antigen specificities of the respective clusters we subjoined established TCR CDR3 sequences reactive with melanoma differentiation (MDA) or cancer testis (CTA) antigen-derived peptide/MHC complexes in silico. These sequences were retrieved from the vdjdb database (https://vdjdb.cdr3.net/; last updated 7th of August 2019) or from a recently published 10 × Genomics dataset (https://support.10xgenomics.com/single-cell-vdj/datasets). In total, we used 106 CDR3 sequences of TCRs recognizing different epitopes of MART-1, thirteen gp100, eight MAGEA1, and six NY-ESO-1. Because some subjoined CDR3 sequences recognizing the same antigen are very similar and thus clustered together, such self-clustering sequences were condensed to one. Finally, the similarity structure of CDR3 sequences was analyzed with the GLIPH algorithm, implemented in R, version 3.5.2 [16].

## Results

### Patients’ history

Four patients with nonresectable metastatic BRAFV600E-mutated melanoma were investigated for changes in the adaptive immune cell tumor infiltrate upon therapy with dabrafenib and trametinib. Patients’ melanoma-specific history is depicted in Fig. 1 and summarized in Table 1. At disease recurrence not amendable by surgery or radiation, patient 2, 3 and 4 were initially treated with immune checkpoint inhibition (ICI), while patient 1 received BRAF/MEK inhibitors as first-line therapy. In detail, patient 2 received the anti-PD-1 antibody pembrolizumab as monotherapy; patients 3 and 4 were treated with the combination of anti-PD-1 and anti-CTLA4 antibodies, i.e., nivolumab and ipilimumab. First-line immunotherapy was discontinued in these three patients because of tumor progression or the occurrence of severe adverse autoimmune reactions (i.e., hypophysitis in patient 3 and colitis in patient 4). The time points of the sequential tumor biopsies are indicated as red asterisks in Fig. 1. The period between the first and second biopsy ranged from 1 month to 1 year. Furthermore, the interval between start of therapy and the 2nd biopsy was 10 months in patient 1, 3 months in patient 2 and 3, and 6 months in patient 4. Furthermore, it should be noted that the second biopsy of patient 1 and 2 were obtained from regressing lesions, whereas in patient 3 and 4 the respective melanoma lesions were progressing.

### BRAF/MEK inhibition increases CD4^+^ and CD8^+^ T-cell infiltration

**Fig. 2:**
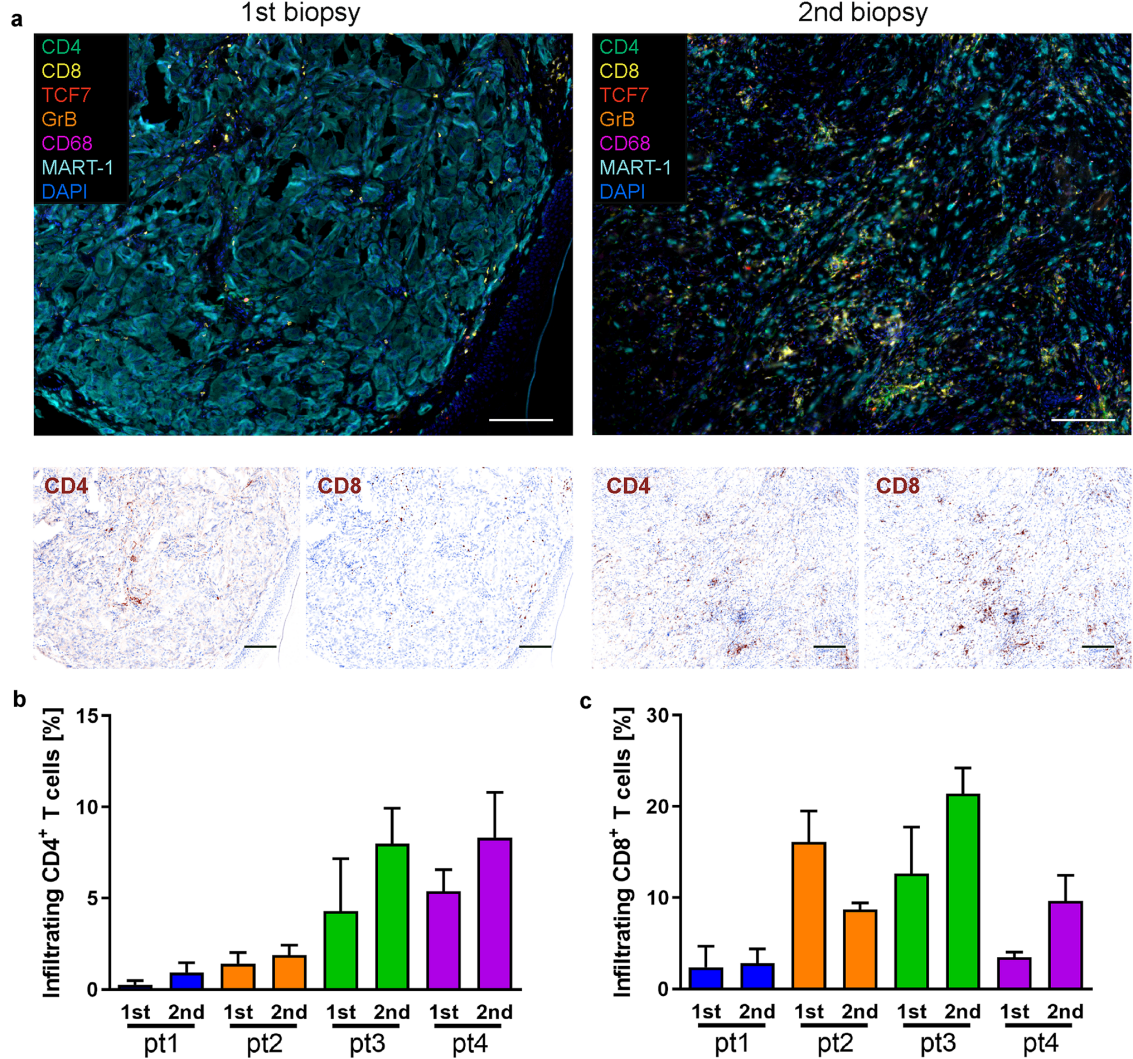
Increased T-cell infiltration upon BRAF/MEK inhibition. **a** An increase in CD4^+^ and CD8^+^ TILs was observed upon BRAF/MEK inhibitor therapy. Multiplexed immunofluorescence staining of melanoma FFPE tissue of patient 3 stained for an immune cell marker panel (CD4—green, CD8—yellow, CD20—red, FOXP3—orange, CD68—purple), melanoma marker (MART-1—light blue) and DAPI (blue); × 10 magnification, scale bar represents 100 μm. The 1st biopsy was taken before therapy and the 2nd biopsy under therapy. CD4 and CD8 staining is depicted as single channel pathology view images (Quantitative Pathology Imaging System (PerkinElmer)); × 10 magnification, scale bar represents 100 μm. **b**, **c** Quantification of CD4^+^ and CD8^+^ TILs of all patients in the 1st and 2nd biopsy by counting and calculating the average infiltration in % of all cells in at least 4 different representative areas (× 20 magnification) of the tumor with the Quantitative Pathology Imaging System (PerkinElmer). Error bars represent + SEM

To visualize changes in the composition of the immune infiltrate within the metastatic lesions caused by BRAF/MEK targeted therapy, we performed multiplexed immunofluorescence staining for CD4, CD8, CD20, CD68, FOXP3, and MART-1 (Fig. 2a and Suppl. Fig. S1). This analysis revealed an increased infiltration of CD4^+^ T cells in all patients (Fig. 2b) and an increased infiltration of CD8^+^ T-cells in 2 out of 4 patients upon therapy (Fig. 2c). However, in patient 1 the T-cell infiltrate even after combined BRAF/MEK inhibition was still confined to the tumor-stroma border (Suppl. Fig. S1a). The number of infiltrating B cells and macrophages was highly variable among patients with no obvious association with therapy (Fig. 2a and Suppl. Fig. S1). In none of the patients, either before or under therapy, we were able to detect relevant numbers of FOXP3^+^ regulatory T cells infiltrating the tumor. There were also some interesting changes with respect to expression of the melanoma differentiation antigen MART-1, which changed from a dispersed cytoplasmic to a perinuclear pattern in patients 1, 2, and 3 (Fig. 2a and Suppl. Fig. S1).

### Kinase inhibitor therapy increases richness and diversity of the T-cell infiltrate

**Fig. 3:**
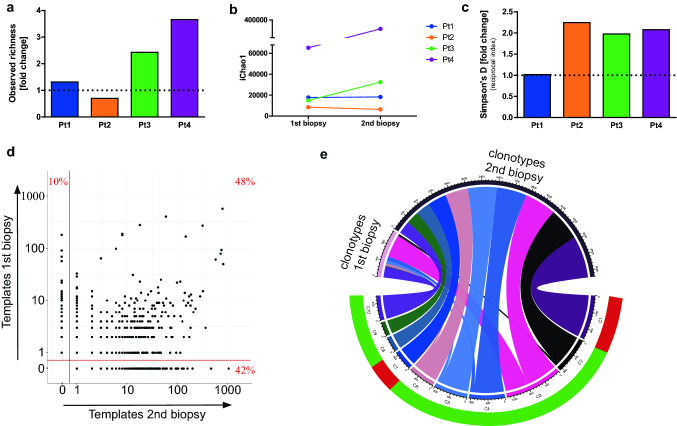
Increased TCR repertoire richness and diversity by expansion of pre-existing T-cell responses. **a**, **b** A clear increase in T-cell infiltration is indicated by the increase in the observed richness reflecting the amount of unique TCR and by the increase of iChao1, which is an estimation of the lower bound of the clonotype richness. **c** The diversity of the TCR repertoire is increased, visible in the increase of Simpson’s diversity index indicating the diversity of the sample by considering the amount and abundance of the TCR clonotypes. **d** Amounts of TCR templates in the 1st and 2nd biopsy of patient 3. The plot demonstrates a shift in the TCR repertoire towards a more abundant immune response upon therapy with many shared clonotypes expanding. Red numbers are fractions of vanishing (upper left quartile), common (upper right region) and newly emerging TCR clonotypes (lower right region). **e** Absolute numbers of templates of the top 10 expanded clonotypes under therapy for patient 3. The absolute numbers are split into the respective numbers of templates from the 1st and 2nd biopsy. The clonotypes that have already been present before therapy are highlighted in green and newly emerging ones in red

To further scrutinize the changes of the T-cell infiltrate in melanoma upon treatment, we examined the respective TCR repertoire by sequencing the highly variable CDR3 region of the TCRβ chains. This analysis demonstrated a strong increase in the T-cell clonotype richness upon therapy in patient 3 and 4 (Fig. 3a). The T-cell clonotype richness represents the number of unique clonotype templates detected in the sample. To estimate the richness of clonotypes with lower abundance, we also applied IChao1, which confirmed the increase in T-cell clonotype richness in patients 3 and 4 and obviated the risk missing an increase in richness for low abundance T-cell clones in patients 1 and 2 (Fig. 3b). Next, we calculated Simpson’s D, which reflects both the number of different clonotypes as well as their respective abundance demonstrating an increased T-cell diversity in 3 of the 4 patients (Fig. 3c).

Increases in T-cell clonotype richness may be due to either recruitment of new T cells or proliferation of pre-existing cells. To discern these alternatives, we compared the individual TCR templates before and under therapy. Figure 3d depicts the number of T-cell receptor templates present in the melanoma lesions before and under therapy separated in T-cell receptor templates that were newly emerging (lower right), vanishing (upper left), or present before and under therapy (upper right). A substantial subset of clonally expanded T cells under BRAF/MEK inhibition were already detectable before therapy in all patients, but patient 4, for whom we also detected a large number of newly expanded T-cell clonotypes upon treatment (Suppl. Fig. S2a). The exact numbers are given in Suppl. Table S1. Quantifying the dynamics of the top 10 expanded TCR clonotypes present in the tumor after BRAF/MEK inhibitor therapy stressed this notion even more (Fig. 3e and Suppl. Fig. S2b). The circle plot shows the overall template number of the top 10 clonotypes under therapy and their respective template number before and under therapy. The exact numbers are given in Suppl. Table S2.

### Induction of TCF7 by BRAF/MEK inhibition

**Fig. 4:**
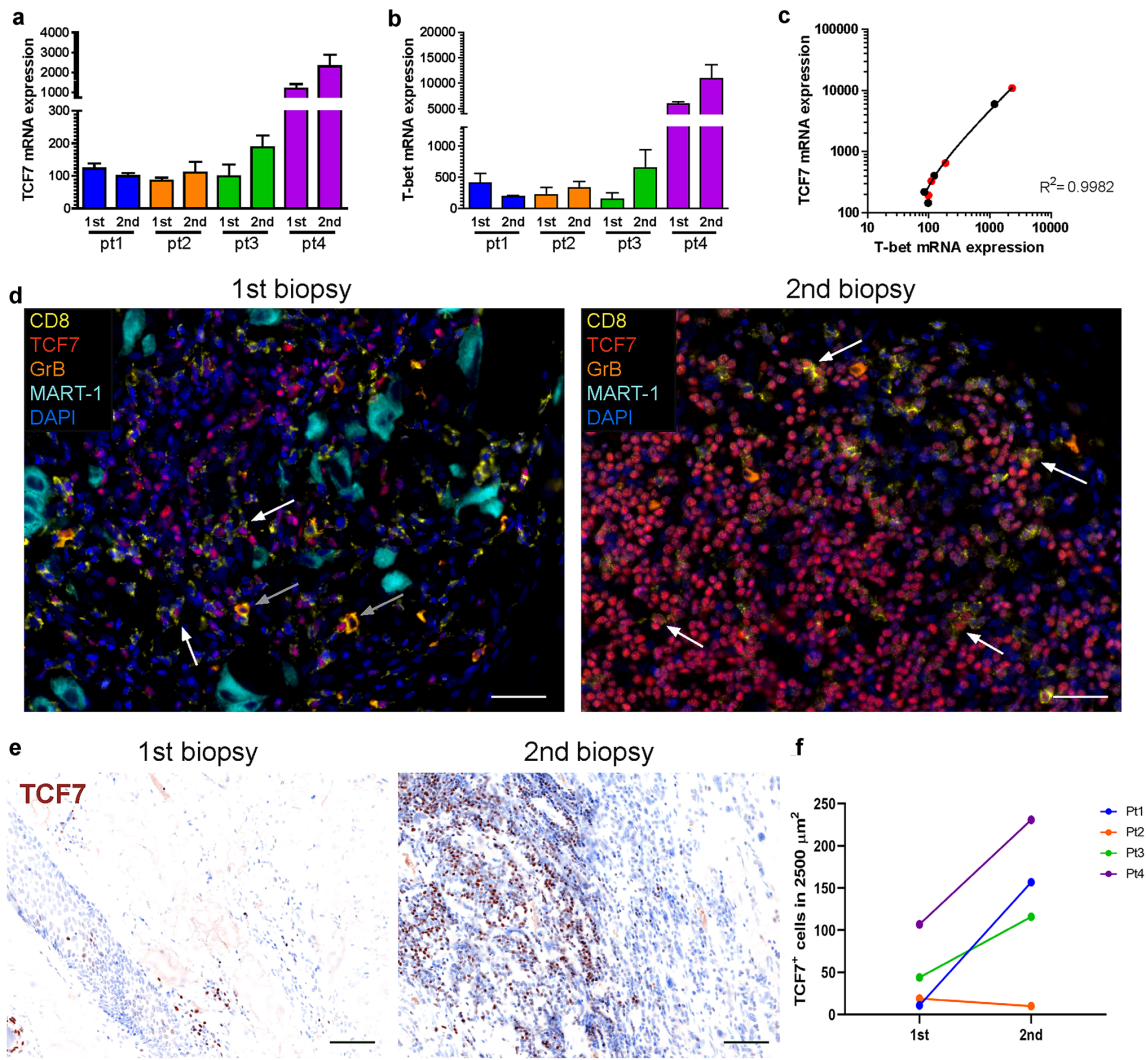
T-bet and TCF7 are upregulated upon BRAF/MEK inhibition. **a, b** TCF7 and T-bet mRNA expression was assessed in the 1st and 2nd biopsy of each patient by qRT-PCR in three different independent experiments and normalized to the RPLP0 housekeeping gene expression. Error bars represent + SEM. **c** Correlation analysis of TCF7 and T-bet expression (*R*^2^ = 0.998). 2nd biopsies are highlighted in red. **d** Multiplexed immunofluorescence of FFPE tissue staining for CD8 (yellow), TCF7 (red), GrB (orange), MART-1 (light blue), and DAPI (blue); × 40 magnification, scale bar represents 25 μm. White arrows mark TCF7 expressing CD8^+^ cells and grey arrow mark GrB expressing CD8^+^ cells. **e** TCF7 staining is depicted as single channel pathology-view images [Quantitative Pathology Imaging System (PerkinElmer)];  × 20 magnification, scale bar represents 50 μm. **f** Quantification of TCF7^+^ cells of all patients in the 1st and 2nd biopsy was performed by counting positive cells in a 2500 µm^2^ representative area, due to the observation that the cells formed dense clusters and were not dispersed over the whole tissue

A plethora of different T-cell subsets with their respective differentiation and activation states has been described among tumor infiltrating lymphocytes (TIL) [17]. Among these, the presence of central memory T cells has been repeatedly reported as a positive predictive marker for response to immunotherapy [16, 18, 19], as these possess a strong ability to expand upon immune checkpoint blockade. Thus, we first examined the levels of mRNA encoding the transcription factors TCF7 and T-bet, which are essential for maintenance of central memory and more differentiated T cells, respectively. Both TCF7 and T-bet mRNA expression was increased in 3 out of 4 patients upon BRAF/MEK inhibition (Fig. 4a, b). Protein expression of TCF7 was confirmed by multiplex immunofluorescence visualizing CD8, TCF7, GrB, and MART-1 (Fig. 4c). Of note, GrB and TCF7 expression was mutually exclusive in CD8^+^ T cells (Fig. 4c, gray arrows). The overall number of TCF7^+^ cells increased upon therapy in 3 of 4 patients (Fig. 4d, e). Notably, TCF7^+^ cells were present in dense clusters and not distributed over the whole tissue.

### MAPK targeted therapy increases the size of TCR clusters with similar reactivity

**Fig. 5:**
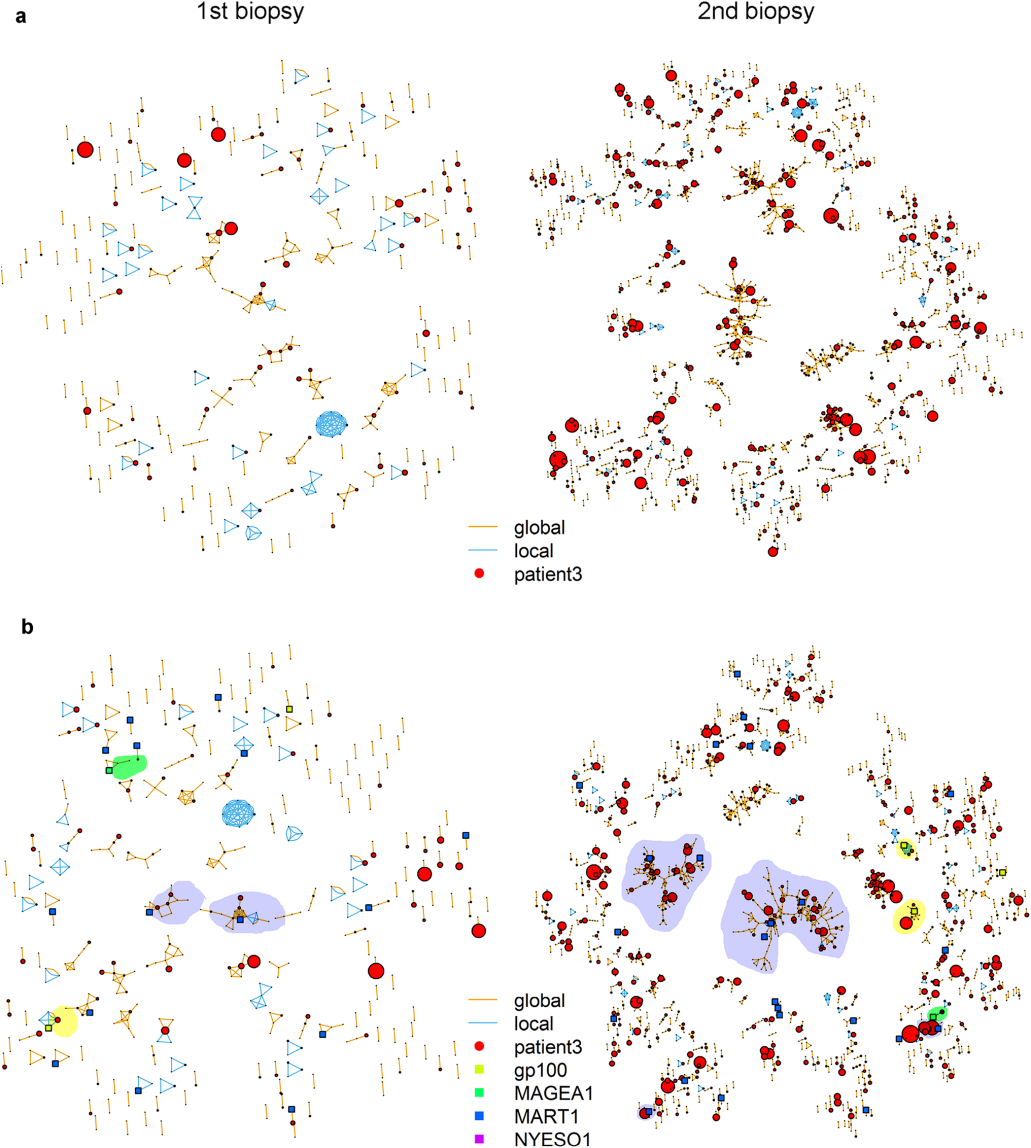
Diverse TCR repertoire forms clusters with similar antigen specificities. **a** The GLIPH algorithm clustered the TCR sequences of patient 3 according to their similarity from the 1st (left) and 2nd (right) biopsy. Every circle represents a TCR sequence of the tumor sample and its size indicates the abundance of the respective T-cell clonotype on a log scale. The circles are connected via blue or orange lines, demonstrating local or global similarity, respectively. Under therapy the number and size of clusters increases. **b** Additional MDA/CTA epitope recognizing sequences were added with fixed abundance values and depicted as squares in different colors. Some larger clusters connected with MDA/CTA recognizing sequences are highlighted in their respective color. Many exist already before therapy and expand during therapys

T-cell reactivity against a given antigen is mediated by T-cells with TCRs that are related at the protein level, but are not necessarily identical. The grouping of lymphocyte interactions by paratope hotspots (GLIPH) algorithm groups TCR amino acid sequences according to their similarity, thus predicting a putative common antigen recognition [15]. Applying this algorithm revealed that the number and size of clustered TCR clonotypes increased and the clonotypes within these clusters became more abundant upon therapy in three out of the four patients (Suppl. Table S3, Fig. 5a). Thus, the observed widening of the TCR repertoire usage is largely caused by a more diverse recognition of a still limited set of antigens. To obtain hints towards the nature of the recognized antigens, we in silico subjoined TCR CDR3 sequences, which have been experimentally verified to recognize melanoma differentiation (MDA) and cancer testis antigens (CTA), i.e., gp100, MAGEA1, MART-1, or NY-ESO-1, derived peptide/MHC complexes (Fig. 5b and Suppl. Fig. S3). Owing to the increased number of sequences and connecting lines, the arrangement of the clusters differs slightly from Fig. 5a. This approach revealed that many MART-1 and some gp100 and MAGEA1 reactive TCR sequences co-clustered with the TCR sequences obtained from the patients’ tumor infiltrates suggesting that the respective cluster is directed against these MDAs/CTAs. Importantly, this notion particularly applies to larger clusters observed under BRAF/MEK inhibition (Fig. 5b and Suppl. Fig. S3).

## Discussion

In the present study, we scrutinized the impact of the combined BRAF/MEK inhibition on tumor infiltrating T cells in melanoma demonstrating an increased presence of CD4^+^ and CD8^+^ T cells upon therapy. This infiltrate was characterized by a more diverse and abundant TCR repertoire usage. Furthermore, the detailed TCR repertoire analyses suggested that the observed increase in CD4^+^ and CD8^+^ T cells resulted from an expansion of T cells present in the tumor before targeted therapy. This expansion was likely facilitated by an induction of the transcription factors TCF7 and T-bet upon BRAF/MEK inhibition. Although TCF7 is involved in self-renewal and persistence of CD8^+^ memory T cells [20], T-bet is crucial for the balance of T-cell memory and effector differentiation as well as for triggering Th1 responses characterized by an IFN-γ signature [21]. Recently, Jansen et al*.* described two distinct T-cell populations necessary for a functioning immune response against human cancer—one consisting of stem-like T cells (characterized by TCF7 expression) and one of more differentiated T cells (characterized by T-bet expression) [22]. Notably, we observed dense aggregates of TCF7^+^ T cells after BRAF/MEK inhibitor therapy (Fig. 4c).

Grouping of lymphocyte interactions by paratope hotspots (GLIPH [15]) revealed two important characteristics of the changed T-cell infiltrate upon BRAF/MEK inhibition: (1) many of the TCRs of expanded T cells cluster together, suggesting that they recognize similar antigens, and (2) several groups co-cluster with TCR sequences with known specificities for shared tumor antigens, i.e., MDAs and CTAs. With respect to the latter, it is important to note that BRAF/MEK inhibition caused an altered MART-1 expression in three out of four patients. While some reports describe an increased MDA expression upon BRAF/MEK inhibition [3], others state a switch towards a more de-differentiated phenotype. Such a de-differentiated phenotype of melanoma cells had been proposed as an immune escape mechanism since it also occurs during the development of resistance to adoptive cell transfer therapy [23]. Thus, in our patients, an enhanced immune response may have caused a de-differentiation of melanoma cells resulting in an altered MART-1 expression. Acquired resistance to targeted therapy cannot be consistently explained by genomic changes. Therapy-induced evolution of tumor and stromal cells has been shown to be at least in part driven by transient transcriptomic changes [24]. Thus, these changes are both complex, interactive, and highly malleable.

The observed effects—induction of a rich and diverse TCR repertoire together with the expression of transcription factors representative for central memory T cells—are in line with the previous reports demonstrating a positive impact of BRAF/MEK inhibitors on the outcome of immunotherapy of melanoma [8, 25], as well as in other cancers [16]. However, the exact mechanisms of these effects on the immune response are not yet fully understood. The inhibition of either BRAF or MEK in melanoma patients is assumed to result in an enhanced tumor antigen expression or the release of these antigens caused by cell death, which is then followed by an increased T-cell infiltration [6, 7, 25]. Thus, the increased infiltrate may be based on either attraction of T cells, or the expansion of a pre-existing T-cell infiltrate [26]. Here, by analysis of sequential biopsies, particularly as these were not the same indexed lesion, we demonstrate that the latter was the case in 3 out of 4 patients.

Immunomodulation by ICI is associated with increased numbers of tumor infiltrating T cells. In difference to the enhanced immune response by BRAF/MEK inhibition reported here, i.e., the expansion of pre-existing T-cell infiltrate, ICI-induced T-cell infiltration of tumors largely depends on the recruitment of cells from the circulating blood [27]. Three patients of the described series received ICI before BRAF/MEK inhibitor therapy. Unfortunately, no sequential biopsies from these patients were available for TCR repertoire analyses. Notably, the observed effects of BRAF/MEK inhibition were comparable to the patient without prior ICI therapy. In patient 2, however, the first biopsy was taken during the ICI treatment, which might explain why the changes in the TCR repertoire in this patient are less pronounced than in the other patients.

As the MAPK signaling pathway is also fundamental for both the priming of naïve T cells as well as their proliferation and survival after cognate activation [2], there is also considerable concern that MEK inhibition may negatively interfere with immunotherapy [28]. In addition, the impact of MEK inhibitors on the further differentiation of T cells into effector and memory T cells is still uncertain. In the presented case series, we observed the induction of transcription factors important for memory and effector T-cell fate. TCF7 has not only been described to promote self-renewal of hematopoietic stem cells [29], but it also maintains stemness in other cell, types such as chondrocytes. Interestingly, MEK inhibition in chondrocytes prevents terminal differentiation via induction of TCF7 [30]. Furthermore, TCF7 positively regulates Wnt signaling, which keeps T cells in a memory stem cell state [31]. Indeed, TCF7-deficient CD8^+^ memory T cells are impaired in their ability to expand upon secondary cognate antigen challenge [20]. Here, we observed that the expression of TCF7 and GrB in CD8^+^ T cells was virtually exclusive, indicating that TCF7 is not expressed in differentiated effector cells, but rather in cells, which still hold a substantial proliferative potential. T-bet is a transcription factor crucial for the differentiation of naïve T cells into an effector phenotype characterized by IFN-γ secretion [21]. Its repression has been observed in poorly functioning T cells in acute myeloid leukemia [32] or chronically infected individuals [33]. Thus, our findings suggest that combined BRAF/MEK inhibition may reverse or prevent terminal differentiation of T-cells by inducing TCF7 and T-bet as well as re-activating Wnt signaling. It should be noted, that this proposed mechanism is not the same as the reinvigoration of “exhausted” T cells by ICI [27]. Hence, the effects of combined BRAF/MEK inhibition in melanoma patients on immune responses should be synergistic or at least additive, thereby explaining the reported clinical benefits by combining immune checkpoint blocking antibodies with targeted therapy [6, 7, 25].

Despite direct effects on effector/helper T cells, BRAF/MEK inhibition may also alter the function and frequency of myeloid-derived suppressor cells (MDSCs) and Tregs [34, 35]. However, due to their rather low and highly variable abundance in the analysed patients we could not really address this notion. Additional indirect effects of BRAF/MEK inhibition are the modulation of the expression of ligands of immune checkpoints or soluble immunosuppressive factors, antigen processing and presentation as well as restoration of DC functionality [6, 36–41].

Limitations of our study include the small patient number, different characteristics of and variable intervals between tumor biopsies. The latter may explain that the changes in TCF7 and T-bet expression were more substantial in patient 3 and 4 than in patient 1 and 2, which were obtained from progressing lesions. Likewise, the time between start of therapy and the second biopsy varies between patients, which may confound our observations. The previous reports suggest that BRAF inhibition leads to an early infiltration of T cells, which, however, decreases again at the time of progression [38]. Others reported that the initial increase in TILs induced by BRAF inhibition absconds within a few weeks [42].

However, even with these limitations in mind, it is important to note that tumor biopsies from patients under BRAF/MEK inhibitor therapy are rarely performed. A notion, which stresses the importance of scrutinizing such biopsies to gain insight into the immunological effects of BRAF/MEK inhibition. Indeed, we believe that this case series provides unique insights into possible immune stimulatory effects of BRAF/MEK inhibitor therapy warranting to be tested in larger cohorts.

In summary, we report a novel facet of the immune-stimulatory effects of BRAF/MEK inhibition on adaptive immune responses in melanoma. Our findings provide a new model suggesting that this therapeutic intervention results in a reprogramming of terminally differentiated T cells by induction of TCF7, thus reinvigorating their proliferative capacity. In line with this hypothesis, we found that these expanding T cells are at least in part specific for MDAs and CTAs. Notably, it was previously reported, that in patients receiving BRAF/MEK kinase inhibitors, the existence of considerable expanded dominant T-cell clonotypes before therapy start, which may have reached terminal differentiation and thus can be reprogrammed, was associated with a favorable therapy outcome [26]. In this regard, it is interesting to note that in patient 2 of our cohort the most dominant clone upon BRAF/MEK inhibition was already present before therapy and this patient showed the best clinical course (CR, Table1). Taken together, despite the fact that this is only a case series, the detailed characterization of the impact of BRAF/MEK inhibition on tumor infiltrating lymphocytes allows an improved understanding of the underlying immunological and molecular mechanisms of targeted therapy in melanoma.

## Electronic supplementary material

Below is the link to the electronic supplementary material.

Supplementary material 1 (PDF 4174 kb)

## Data Availability

Data are available upon reasonable request.

## Abbreviations

AKT: Protein kinase B
BRAF: B-rapidly accelerated fibrosarcoma
CDR3: Complementarity determining region 3
CTA: Cancer testis antigen
ERK: Extracellular signal-regulated kinase
FFPE: Formalin-fixed paraffin-embedded
GLIPH: Grouping of lymphocyte interactions by paratope hotspots
GrB: Granzyme B (protein)
ICI: Immune checkpoint inhibition
MDA: Melanoma differentiation antigen
MEK: MAPK/ERK Kinase
TBX21/T-bet: T-box transcription factor 21
TCF7: Transcription factor 7

## Footnotes

1 https://www.fda.gov/drugs/resources-information-approved-drugs/fda-approves-atezolizumab-braf-v600-unresectable-or-metastatic-melanoma; Accessed 5.09.2020.

## Code availability

Bioinformatic approaches are described in detail in the materials and methods section.
