# Supplementary material for: BRAF and MEK inhibition in melanoma patients enables reprogramming of tumor infiltrating lymphocytes
Source: Cancer Immunol Immunother. 2020 Dec 4;70(6):1635–47. doi: 10.1007/s00262-020-02804-4 (PMC8139931; doi:10.1007/s00262-020-02804-4)
Supplement: Supplementary file 1 — Supplementary material 1 (PDF 4174 kb) [file 262_2020_2804_MOESM1_ESM.pdf]

Suppl. Fig. S1

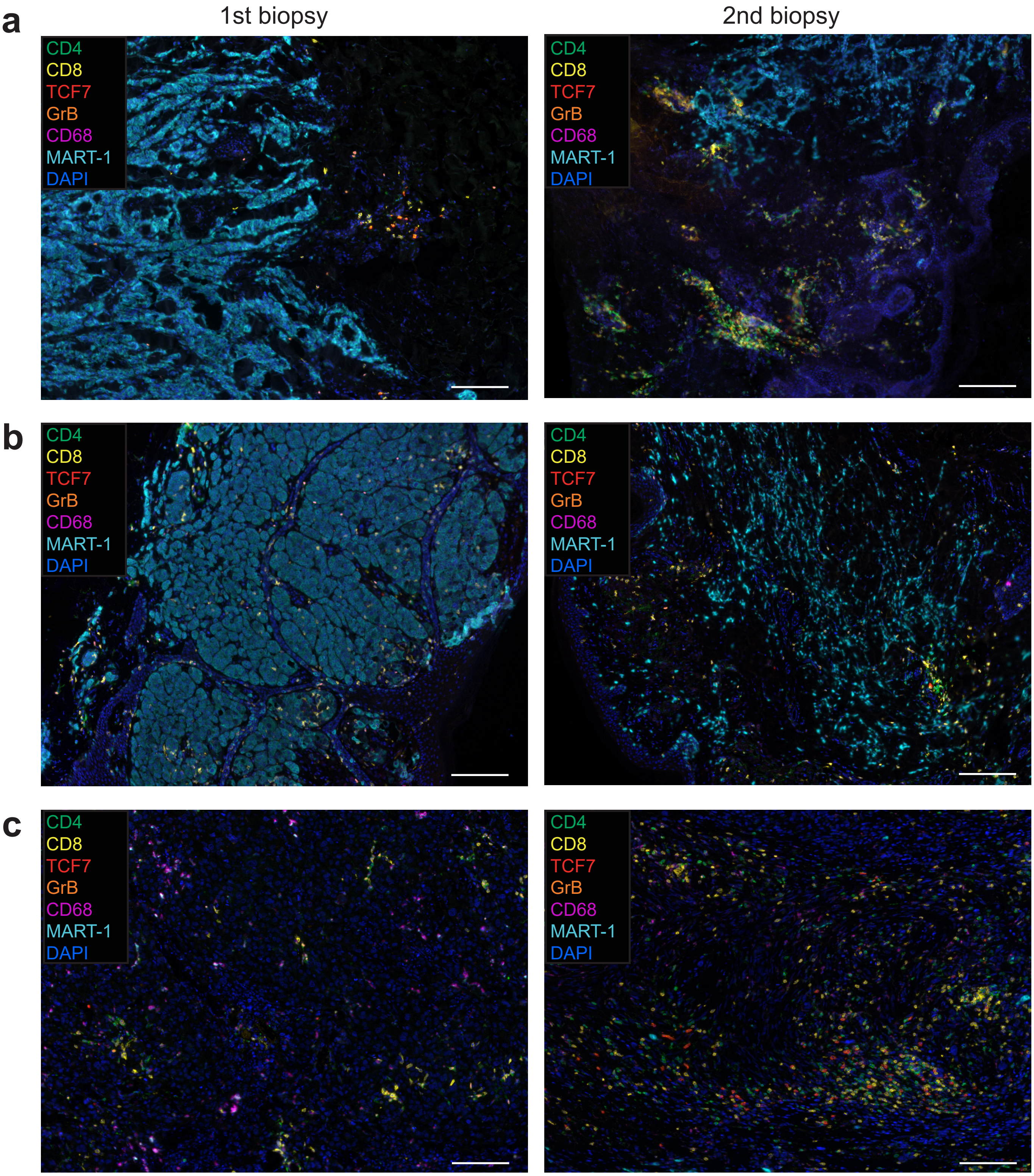

Suppl. Fig. S1. Increased T-cell infiltration upon BRAF and MEK inhibition. An increase of $CD4^+$ and $CD8^+$ TILs was observed upon BRAF and MEK inhibitor therapy. Multiplexed immunofluorescence staining of melanoma FFPE tissue of patient 1 (**a**), 2 (**b**) and 4 (**c**). The 1st biopsy was taken before therapy and the 2nd biopsy under therapy. Stained for an immune cell marker panel (CD4 – green, CD8 – yellow, CD20 – red, FOXP3 – orange, CD68 – purple), melanoma marker (MART-1 – light blue) and DAPI (blue); 10x magnification, scale bar represents 100 µm.

Suppl. Fig. S2

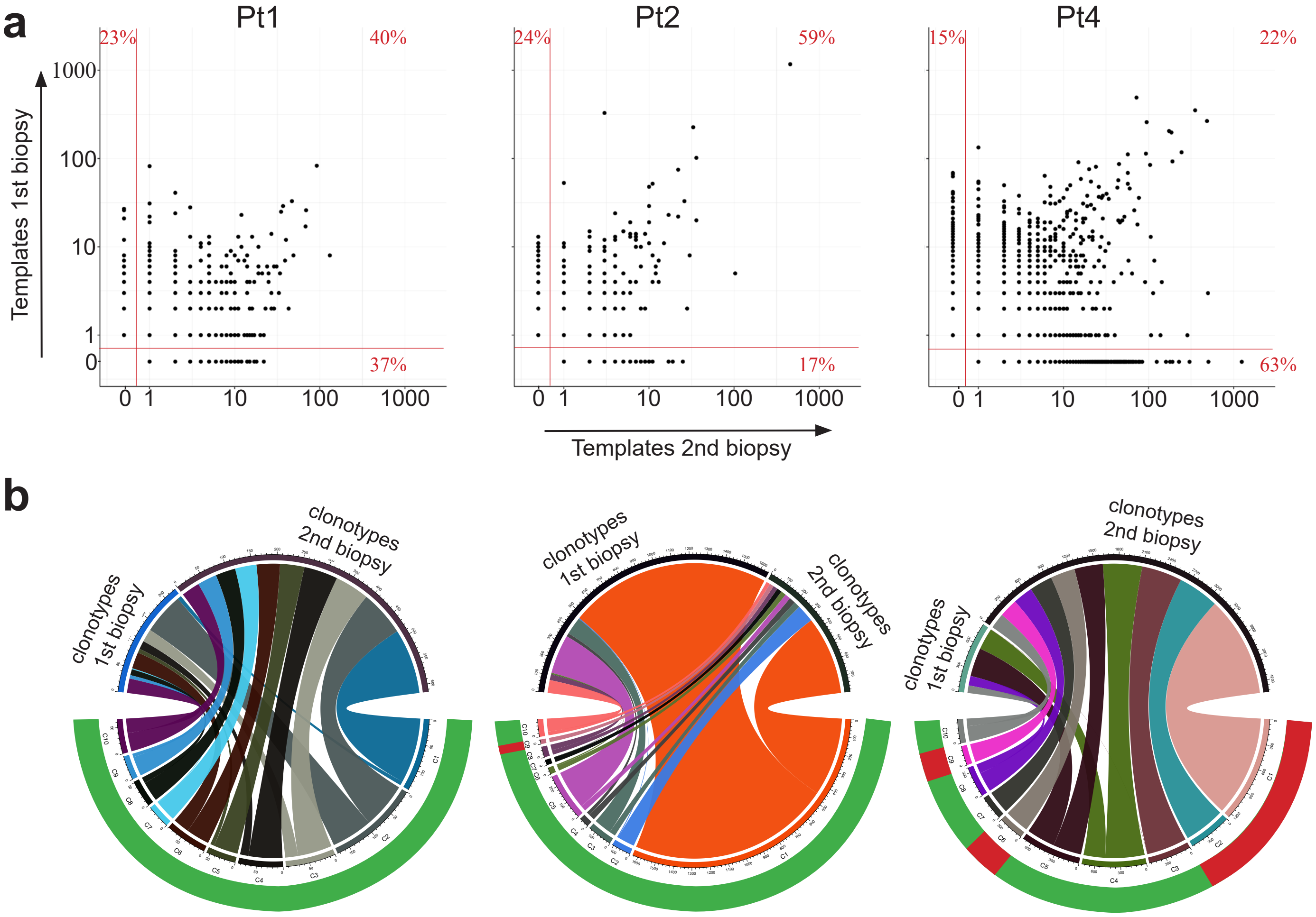

Suppl. Fig. S2. Increased TCR repertoire richness and diversity by expansion of pre-existing T-cell responses. a Amounts of TCR templates in the 1st and 2nd biopsy of patient 1, 2 and 4. Plots demonstrate a shift in the TCR repertoire towards a more abundant immune response upon therapy with many shared clonotypes expanding. Red numbers are fractions of vanishing (upper left quartile), common (upper right region) and newly emerging TCR clonotypes (lower right region). **b** Absolute numbers of templates of the top 10 expanded clonotypes under therapy for patient 1, 2 and 4. The absolute numbers are split into the respective numbers of templates from the 1st and 2nd biopsy. The clonotypes that have already been present before therapy are highlighted in green and newly emerging ones in red.

Suppl. Fig. S3

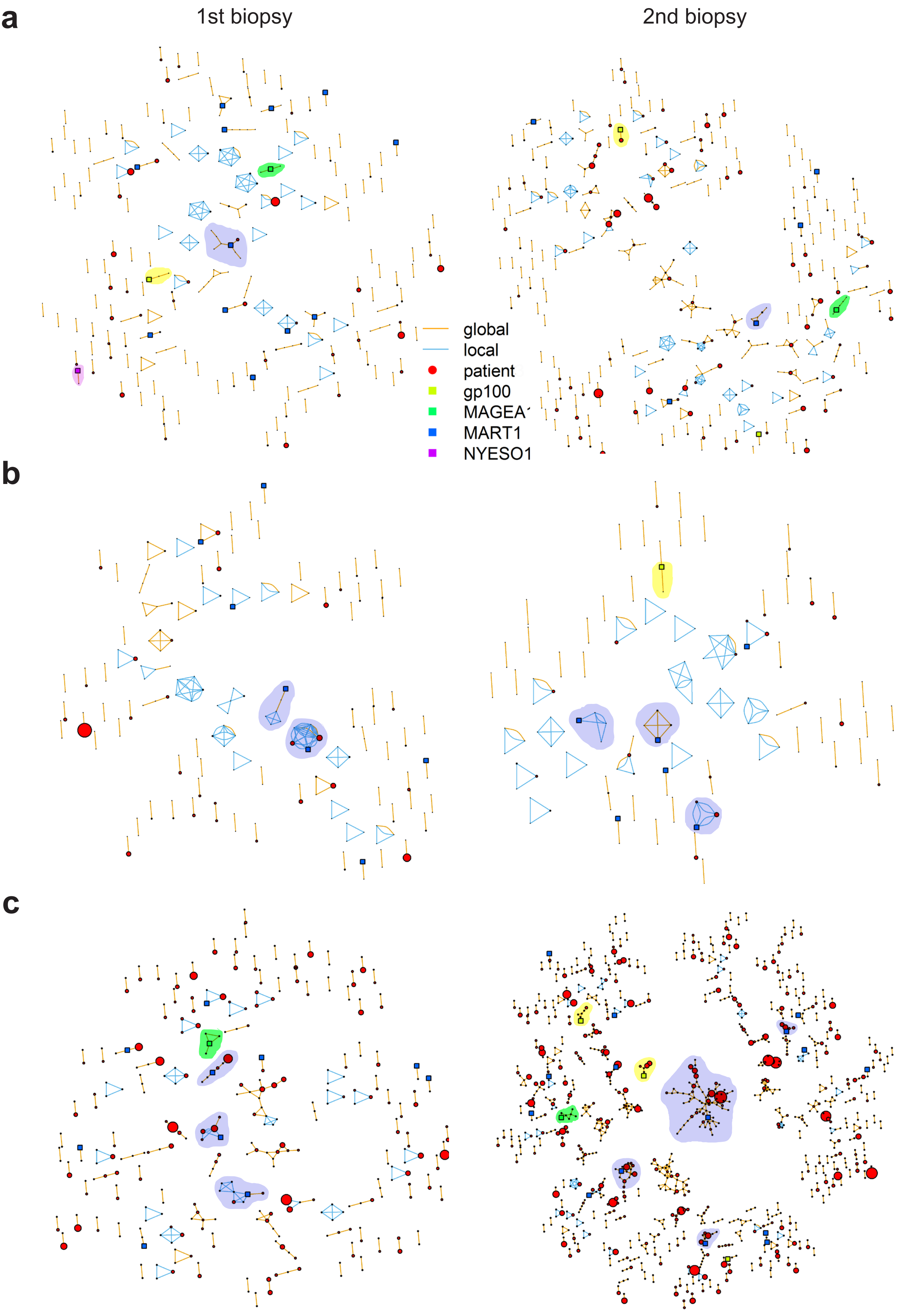
a
1st biopsy
2nd biopsy
global
local
patient
gp100
MAGEA
MART1
NYESO1
b
c

Suppl. Fig. S3. Diverse TCR repertoire forms clusters with similar antigen specificities. The GLIPH algorithm clustered the TCR sequences of patient 1 (**a**), 2 (**b**) and 4 (**c**) according to their similarity from the 1st (left) and 2nd (right) biopsy. Every circle represents a TCR sequence of the tumor sample (red) and its size indicates the abundance of the respective T-cell clonotype on a log scale. The circles are connected via blue or orange lines, demonstrating local or global similarity, respectively. Under therapy the number and size of clusters increases. Additional MDA/CTA epitope recognizing sequences were added with fixed abundance values and depicted as squares in different colors. Some larger clusters connected with MDA/CTA recognizing sequences are highlighted in their respective color. 36 Many exist already before therapy and expand during therapy.
